# Diversity, Distribution, and Plasmodium falciparum Sporozoite Infectivity of Anopheles Mosquitoes in Flood-Prone Communities of Kasese District, Western Uganda: A Cross- Sectional Household Entomological Study

**DOI:** 10.21203/rs.3.rs-11062919/v1

**Published:** 2026-10-11

**Authors:** Rogers Kalyetsi, Kaylin Lewandowski, Ronnie Ndizeye, Raquel Reyes, Mbusa Rapheal, Abel Bwambale, Emmanuel Baguma, Liao Huipeng, Dana A. Giandomenico, Bonnie E Shook-Sa, Moses Ntaro, Peter Chris Kawungezi, Hope Mudondo, Charles Nkubi Bagenda, Paul E. Alele, Jerome Kabakyenga, Gertrude N. Kiwanuka, Edgar M. Mulogo, Ross M. Boyce, Michael H. Reiskind

**Affiliations:** Mbarara University of Science and Technology; North Carolina State University; Mbarara University of Science and Technology; University of North Carolina at Chapel Hill; Mbarara University of Science and Technology; Mbarara University of Science and Technology; Mbarara University of Science and Technology; University of North Carolina at Chapel Hill; University of North Carolina at Chapel Hill; University of North Carolina at Chapel Hill; Mbarara University of Science and Technology; Mbarara University of Science and Technology; Mbarara University of Science and Technology; Mbarara University of Science and Technology; Mbarara University of Science and Technology; Mbarara University of Science and Technology; Mbarara University of Science and Technology; Mbarara University of Science and Technology; University of North Carolina at Chapel Hill; North Carolina State University

**Keywords:** Sporozoite infectivity, Flood-prone villages, malaria vector, Anopheles, Uganda

## Abstract

**Background:**

Malaria poses an ongoing public health challenge for children in Uganda, especially in climate-vulnerable, flood-prone communities. Understanding malaria vector species diversity and transmission measures is vital to guide vector control in these unique environments. We sought to determine the vector species diversity, distribution, and *Plasmodium falciparum* sporozoite infectivity in flood-prone villages in the Kasese district, Uganda.

**Methods:**

We conducted a cross-sectional study in which we collected adult mosquitoes indoors and outdoors from 154 consented sentinel households using unbaited CDC light traps. Mosquitoes were identified morphologically to genus and species. Adult female *Anopheles* spp. were tested for *P*. *falciparum* circumsporozoite antigen by ELISA. We estimated associations between entomological outcomes and covariates of interest, including place of residence, net ownership, household location and construction materials, and reported history of floods in the previous year.

**Results:**

Between 19th February and 22nd May 2025, a total of 1,772 mosquitoes were collected in 136/154 (88.3%) households. Adult *Anopheles* mosquitoes were collected in 54/154 households (prevalence 35.1% (95% CI: 27.9–43.0)). The highest presence of *Anopheles* mosquitoes was in Karusandara (58%), and the lowest in the more urban municipality area (22%). We documented diversity in known malaria vectors, with *Anopheles gambiae s.l*. (73.7%), and *An. funestus* (9.4%) being most common, but also *An. pretoriensis, An. rivulorum, An. mouchetti, An. nili, and An. marshalli* were identified. *Plasmodium falciparum* circumsporozoite antigen was detected from three female *An. funestus* demonstrating a mosquito sporozoite infectivity rate of 1.49% (03/202) (95% CI: 0.48–4.53), and a household positivity rate of 1.95% (3/154) (95% CI: 0.62–5.91) across the study area.

**Conclusion:**

The varied species diversity may sustain malaria transmission in this region of rapidly changing ecological conditions as a result of flooding. The sporozoite infectivity and household positivity emphasize ongoing transmission of malaria, albeit at a low levels, even during an atypically dry period. There is a need to strengthen implementation of targeted vector control interventions with a focus on flood-prone and low-altitude communities within Kasese District, western Uganda.

## Background

Malaria is a global health challenge, especially in sub-Saharan Africa (1, 2). Uganda is among the most highly burdened countries, contributing approximately 5.1% to global morbidity and 2.9% of mortality (1). Malaria is endemic in more than 90% of Uganda, with varied transmission intensity across different regions of the country (3), where it accounts for 30 − 50% of outpatient visits (1), and half of paediatric inpatient deaths (4). Environmental factors such as altitude, proximity to water bodies, rainfall, and flooding events are significantly associated with mosquito breeding, abundance, and transmission rates (5). These associations are also seen in Kasese district, south-western Uganda, where altitude, climate, flooding events, and proximity to flood-prone water bodies has a documented impact on malaria epidemiology (6). Similar environmental determinants have been reported in Kenya and Ethiopia to influence vector distribution (5).

The primary vectors of malaria in Uganda are *Anopheles gambiae* and *Anopheles funestus* (7). In addition, other potential malaria vectors include *An. nili*, *An. mouchetti*, *An. hancocki*, *An. rivulorum*, *An. pharoensis*, and the geographically restricted *An. bwambae* (7, 8). Because these species differ in their ability to transmit malaria and their ecology, understanding vector species composition, abundance, and transmission dynamics in settings where malaria transmission is stable is essential for effective malaria prevention and critical for malaria elimination. This is especially important in areas with low transmission, where there may be a shift from domestic to peridomestic transmission (9).

Persistence of malaria transmission in flood-prone settings without routine and intensified vector control may worsen the clinical treatment outcomes among vulnerable groups. There are gaps in localised entomological surveillance in ecologically heterogeneous flood-prone communities that need to be addressed to enact sustainable malaria control.

### Objectives

We aimed to estimate household-level prevalence of adult mosquitoes, describe *Anopheles* species composition, and measure mosquito-level and household *Plasmodium* sporozoite positivity across flood-prone sub-counties and river catchments in Kasese District, western Uganda. We hypothesized that the presence of adult *Anopheles* mosquitoes would be higher in lower-elevation households, in households closer to flood-prone rivers, and in households with recent flooding or older/damaged LLINs.

## Methods

### Study setting and design

We conducted a cross-sectional entomological study between 19th February and 22nd May 2025 in selected flood-prone villages across Kasese district, western Uganda. The district spans a rural, highland area of approximately 2,700 km^2^, which is characterized by deep river valleys and steep hillsides with elevations of up to 5,000 m. The equatorial climate of the Kasese District allows for year-round malaria transmission, with peaks in January and July that correspond to periods of increased precipitation (6, 10). This district was selected purposely based on reported malaria epidemics and history of floods in recent years (11). Adult mosquito sampling was conducted in ten (10) of the 39 Sub Counties. The Sub Counties were selected based on the presence of flood-prone rivers, history of floods, proximity to flood-prone rivers (Not more than 3 km from the rivers Nyamwamba, Mubuku, and Nyamugasani), and the malaria burden (Fig. 1). Within the 10 Sub Counties, we selected 154 sub-villages, which averaged 44.6 households and 197 people. Each sub-village was under the supervision of a trained village health worker officially designated as a Village Health Team (VHT) member. The selection criteria for sub-villages within Sub County were based on ecological and demographic characteristics such as landscape, vegetative cover, areas of high human density, history of flooding, and proximity to flood-prone rivers. One household from each of the 154 sub-villages was selected. For each sub-village, the team approached the closest house to the designated central reference point, using the local VHT as a guide and asked the head of household or other adult if they would take part. If the selected house declined to take part, then the next closest house was approached, and this was repeated until one willing household from each sub-village was recruited.

### Participant recruitment and enrollment procedures

The research teams held meetings with Sub County and village leaders in all the respective areas during the pre-implementation phase to ensure shared understanding and discussion of the objectives, study design, and implementation procedures. Feedback from these discussions guided the field implementation plan, including designing community mobilization, household access, country-specific communication strategy, and liaison with Village Health Teams (VHT). On the day of data collection, medical entomology technicians, accompanied by data clerks, visited the eligible households in the sub-village together with their respective VHT to carry out the survey and mosquito collection. We excluded non-residential structures, unoccupied dwellings, and households where trap placement could not be performed safely. An adult household head (≥ 18 years) or their delegated representative was informed about the study objectives and procedures in the local language (Lukhonzo) by a trained study staff member fluent in Lukhonzo and invited to provide written informed consent for voluntary participation, including permission for the research team to enter the household and compound to collect adult mosquitoes. After obtaining written informed consent, household location was geolocated using handheld GPS devices. Household characteristics (Table 1) were recorded in an electronic database (12, 13), using a structured household questionnaire/data-collection guide administered at the time of consent. A systematic method of household survey and mosquito collection was followed across each study household (14, 15); to reduce variations and potential biases in data collection and the measured outcome.

### Surveillance for adult mosquitoes

Adult mosquitoes were collected indoors and outdoors once between February and May 2025 using battery-powered CDC light traps (16) (Model 512; John W. Hock Company, Gainesville, FL, USA) from each sentinel household. Traps were set every evening between 17:30 and 18:30 hours, when adult mosquitoes’ feeding activity starts peaking, and were picked the following morning between 07:00 and 08:30 hours. Indoor resting adult mosquitoes were also collected every morning using a battery-powered Prokopack aspiration system (17). The outdoor traps were set in tree branches at a height of approximately 2 m off the ground, and indoor traps were put near the sleeping spaces at the foot end of the bed at approximately 70 cm from the ground (18). Labeled collection cups were used to collect mosquitoes. As soon as the collection was completed, the lid was put tightly on the collection cup before turning off both systems. The cups were detached from the traps and the aspirator and transferred to the field station laboratory for cold knockdown using the refrigerator before sorting and processing (19). All captured arthropods were sorted into separate adult mosquitoes. The adult *Anopheles* mosquitoes were then counted, sexed, and identified to species using standard morphological identification keys (20–22). Adult mosquitoes were preserved dry in labeled vials containing silica gel and organized by household, collection site, and date of collection. Vials were punctured at the top to prevent moisture buildup and were small enough to prevent silica gel from being lost. All specimens were then stored at Mbarara University of Science and Technology Clinical and Research Laboratory for further processing. Identified mosquitoes were shipped to North Carolina State University, United States of America, for *P. falciparum* sporozoite infectivity rate testing using a circumsporozoite protein enzyme-linked absorbent assay (CS-ELISA) as described below.

### Mosquito sample preparation and sporozoite infectivity testing

Mosquito samples were prepared a day before the CS-ELISA was performed (23, 24). Using a scalpel, the mosquito head and thorax were carefully dissected from the abdomen (25). A single mosquito head and thorax were placed into a labeled 1.5 mL microcentrifuge tube containing 50 μL of IGEPAL CA-630 grinding/blocking buffer and homogenized thoroughly using a sterile pestle. The pestle was rinsed twice with 100 μL of grinding buffer, with each rinse added to the homogenate to obtain a final volume of approximately 250 μL. The homogenate was stored at 4°C for up to 24 hours or at − 20°C before processing the following day. To prevent cross-contamination, the pestle was rinsed twice with PBS-Tween and dried with clean tissue paper between processing individual mosquitoes.

The presence of *P. falciparum* sporozoites was determined using an ELISA reagent kit based on the dual monoclonal antibody sandwich principle that identifies the presence or absence of specific peptide epitopes of circumsporozoite (26). The *Plasmodium falciparum* Sporozoite ELISA Reagent Kit, MRA-890, Atlanta, Georgia, USA (24) was used following an already published protocol (27). All samples that tested positive in the screening phase were boiled at 100°C on the hot block for 10 minutes and re-tested for circumsporozoite proteins to eliminate false positives caused by heat-unstable cross-reactive proteins (28). Only samples that tested positive during the screening and after boiling were considered positive (28).

### Outcomes

The primary outcomes were: (1) household adult mosquito presence, defined as collection of at least one adult mosquito inside or outside; (2) household *Anopheles* presence, defined as collection of at least one adult *Anopheles* mosquito inside or outside; (3) household *Anopheles* count inside or outside; (4) *Anopheles* species composition inside or outside. Because of low numbers of mosquitoes, we combined all mosquito trapping approaches for each household; (5) mosquito-level *P. falciparum* circumsporozoite positivity among tested female *Anopheles* specimens; and (6) household-level sporozoite positivity, defined as at least one sporozoite-positive *Anopheles* from that household. Covariates of interest included sub-county, river catchment, distance from riverbank, terrain/elevation, household size, LLIN ownership, number of LLINs, LLIN age, and LLIN physical condition, and history of flooding, water source, livestock ownership, and house-construction/screening characteristics.

### Data management and analysis

Data management was conducted using REDCap, and all statistical analyses were done using STATA version 17. Descriptive data analyses included frequencies and percentages with corresponding 95% confidence intervals, stratified by Sub County and river catchments. Associations between mosquito outcomes (presence/absence) and covariates of interest, including place of residence (sub-county), ownership of LLIN, age of LLIN, presence of windows, eaves, terrain, distance from the riverbanks, and reported history of floods in the previous year, were estimated. Statistical significance was defined at α = 0.05. The *P. falciparum* sporozoite infectivity rate at the mosquito and household levels was estimated as proportions with 95% exact binomial confidence intervals (29). We excluded mosquitoes not tested for sporozoite infectivity because they did not have complete information at the time of analysis.

### Ethical Considerations

The study was approved by the Mbarara University of Science and Technology Research Ethics Committee (MUST-2024–1538), the Uganda National Council for Science and Research (HS4978ES), and the University of North Carolina at Chapel Hill (24–0233). Written informed consent was obtained from the adult household head or delegated adult representative before household data collection and mosquito sampling

## Results

### Participant flow chart

One hundred sixty households were assessed for eligibility for entomological surveillance across flood-prone Sub Counties in Kasese District (Fig. 2). Of these, 154 households were enrolled and sampled between February and May of 2025. Six households were excluded for lack of consent or unavailability of household members at the time of surveillance.

### Household-level distribution of adult mosquito genera based on household and environmental characteristics

At least one adult mosquito of any genus was collected from 136 of the 154 households, corresponding to a household mosquito presence of 88.31% (95% CI: 82.15–92.54) (Table 1). Adult mosquito presence was slightly higher among households without LLIN than among those owning at least one LLIN (90% vs. 87%, Chi-square test, p = 0.58), although the difference was not statistically significant. Similarly, mosquito presence was higher among households without screened windows (94% vs. 83%, Fisher’s exact test, p = 0.06), and among households without screened ventilators than those with screened ventilators (90% vs. 80%, Fisher’s exact test, p = 0.25), although the differences were not significant.

#### Distribution of Anopheles mosquitoes across household and environmental characteristics

Among the households sampled, 54/154 had at least one *Anopheles* mosquito (presence 35.1% (95% CI: 27.9–43.0)) (Table 2). Households with many old LLINs were linked to a higher presence of adult mosquitoes compared to households with few old LLINs (> 5 years 71% vs. < 2 years 24%, Fisher’s exact test, p = 0.03). Similarly, A*nopheles* mosquitoes were more common in households with older LLINs (> 1 year) compared to those with newer LLINs (< 1 year) (old, 24% vs. new 9%, Fisher’s exact test, p = 0.04).

#### Species composition and distribution of Anopheles mosquitoes in the study area

Overall, 213 *Anopheles* specimens were identified morphologically (Table 3). *An. gambiae s.l*. was the most predominant species, 73.7% (157/213), followed by *An. funestus*, 9.4% (20/213), and the least was *An. marshalli* species, 0.9% (2/213). Overall, the composition of *Anopheles* species varied across the study sub-counties and households. Karusandara Sub County recorded the greatest species diversity, with five *Anopheles* species detected. *Anopheles* mosquitoes were detected in 58% of surveyed households in Karusandara, *with An. gambiae s.l* being the predominant species (Table 4). Kyarumba Sub-county had four *Anopheles* species identified from 40% of surveyed households. In Kisinga Town Council and Kasese Municipality, fewer *Anopheles* species were recorded compared to Karusandara and Bulembya, although *An. gambiae* s.l remained the predominant species across all locations (Table 5).

### Plasmodium falciparum sporozoite infectivity in adult Anopheles mosquitoes

Overall, 213 *Anopheles* specimens were identified morphologically, of which 202 were tested for *P. falciparum* circumsporozoite antigen. Eleven were not tested due to either the lack of a positive identification because of specimen condition or being male. Three female *An. funestus* mosquitoes tested positive (3/202), corresponding to a sporozoite infectivity of 1.49% (95% CI: 0.48–4.53). The positive adult female *Anopheles* mosquitoes originated from three different households (3/154), giving a household positivity of 1.95% (95% CI: 0.62–5.91). Of the three infected mosquitoes, two were collected from two households (one each, from Karusandara Sub County and Ibanda Town Council) along the Mubuku River catchment. The third was collected from a household in Kisinga Town Council along the Nyamugasani River catchment.

## Discussion

This study examined the distribution, species composition, and infectivity of *Plasmodium falciparum* in *Anopheles* mosquitoes within flood-prone communities of Kasese District, Uganda. A noteworthy finding of this survey is the unexpected inverse relationship between *Anopheles* presence and bed nets. Specifically, households with more, but older bed nets were more likely to have an *Anopheles* mosquito present than households with fewer, but newer bed nets. This finding was limited to *Anopheles* mosquitoes. This counterintuitive finding likely reflects both the size of the household and residents’ awareness that the risk of malaria is high, so more are likely to have and use bed nets, which may correlate with *Anopheles* presence. Age of bed net was also significant, with older bed nets associated with a greater likelihood of *Anopheles* being present. This is a more logical finding, as bed nets impregnated with insecticide lose their effectiveness over time. As noted above, the presence of bed nets suggests both prior and likely ongoing risk of malaria and while newer nets have control effectiveness, old nets do not.

There was some heterogeneity of species across different sub-counties in malaria vectors, reflected by seven identified species. *Anopheles gambiae s.l*. was the most prevalent species (65–88% of *Anopheles* caught), followed by < 20% *An. funestus*. The presence of *An. funestus* and other secondary species indicates potential for complementary or residual transmission, enhancing malaria resilience, particularly post-flooding when breeding sites increase. The detection of *An. funestus*, although comprising less than 20% of captures, is significant due to its high vector capacity, competence, longevity, and indoor feeding and resting habits (30).

These findings are consistent with broader regional observations from the Kenyan highlands, where *An. gambiae s.l* was the predominant species, followed by *An. funestus*, indicating the two as the main vectors of malaria (31, 32). Contrary to these observations, studies in lowland sites in similar studies showed *An. funestus* as the dominant species (31), reflecting the role of larger, more permanent habitats and existing vector control strategies (LLIN distribution and use) influencing the abundance of *An. funestus* populations (33). Additionally, the presence of various secondary *Anopheles* species in the study area suggests the likelihood of outdoor malaria transmission, not fully addressed by indoor-focused interventions (34, 35).

*Plasmodium falciparum* malaria is the leading cause of morbidity across Uganda (8). The *P. falciparum* sporozoite infectivity of 1.49% (03/202), restricted to *An. funestus*, and the household positivity of 1.95% is consistent with the observed 3.3% malaria prevalence in the same settings, indicating moderate to low transmission levels (33, 36). However, the low infectious rate of the vector could sustain malaria transmission risk in vulnerable populations across Kasese district (37).

The lack of *Pf* sporozoite-positive *An. gambiae*, and exclusive detection of Pf sporozoite positive *An. funestus* in these flood-prone communities may be explained by contrasting breeding habits and longevity (38, 39). *Anopheles funestus* prefers relatively large permanent water pools, vegetated fresh-water sites, and slow-flowing streams for breeding(39). In contrast, *An. gambiae* breeds in smaller temporary pools of exposed water, and *An. funestus* has greater and increased persistence in both wet and dry seasons compared to that of *An. gambiae*(38). Following flood recession, permanent vegetated pools and drainage channels remain, where sustained *An. funestus* breeding can persist (39, 40). Similar observations have been reported in related studies, with *An. funestus* a more likely vector in areas with flooding (39, 41–44). As a result, the predominance of *An. funestus* positive for sporozoites suggests prolonged malaria risk for extended periods following flooding. The findings highlight the need for heightened species-specific surveillance, sporozoite screening, testing of insecticide susceptibility, and vector control interventions, which continue to be effective for *An. funestus* within the immediate post-flood period. Though no *An. gambiae* tested positive for sporozoites, it is still likely they might still play a role in transmission particularly during periods with more abundant rainfall.

The sporozoite rates are consistent with others from moderate transmission settings in East Africa: eastern Uganda (Tororo- Busia) 1.31% (45), 0.9% in Ethiopia (46), and 2.0% in Kenya (37). However, contrary to our findings, higher sporozoite positivity rates were observed in other studies: 4.6% in Tanzania (47), 9.5% in Kenya (48), and 3.2% in Tororo, Uganda (33). The differences could reflect variations in seasonal malaria transmission intensity, vector abundance, implementation of vector control interventions, and differences in sampling methods used. For instance, our study used CDC light traps that are associated with low yields of *Anopheles* mosquitoes compared to human landing catches or larger surveys (49, 50), thus under-sampling hot spots. In addition, this study was implemented during an un-seasonally dry period and two years after a distribution campaign of LLINs in the area. The survey was done prior to flooding events, which are associated with low mosquito densities relative to post-flooding (51, 52).

### Study strengths and Limitations

The study’s strongest feature was its field-based entomological surveillance in Kasese District, Uganda, encompassing three major river catchments and ten sub-counties with diverse and flood-prone environments. Utilizing standard CDC light traps and aspiration methods for indoor and outdoor mosquito sampling ensured consistent measures of mosquito densities among sentinel households. By integrating entomological, household, environmental, spatial, and parasitological data, the study offered a comprehensive overview of malaria vector distribution and transmission potential. Morphological techniques were used to identify mosquito species, and CS-ELISA tests confirmed the presence of infectious mosquitoes, with a heating step to eliminate false positives. These combined methods yielded reliable entomological data in a neglected flooding-prone area.

This study utilized a cross-sectional design to collect mosquitoes in a fixed surveillance period, limiting insights into seasonal or post-flood variations in vector abundance and infection rates. The use of unbaited CDC light traps and indoor aspiration led to an underrepresentation of outdoor, exophagic species, potentially skewing results toward indoor-resting species. Species identification was based solely on morphological features, preventing the differentiation of sibling species within complexes like *An. gambiae s.l*. and *An. funestus*. Additionally, the dry conditions preceding the collection may not reflect typical environmental factors affecting vector transmission, and the sentinel household method does not provide a probability sample of all households in Kasese District, affecting the generalizability of findings.

### Conclusion

Varied species diversity in flood-prone communities may play a crucial role in sustaining malaria transmission between distinct rainfall periods. Longitudinal studies are needed to document shifts in mosquito populations, but control programs may need to consider targeting the diverse ecologies and behaviors of these less common species. Interventions conducted during periods between flooding, when mosquito populations and infectivity rates are low, could yield exponential gains, similar to that seen with SMC programs.

## Figures and Tables

**Figure 1:**
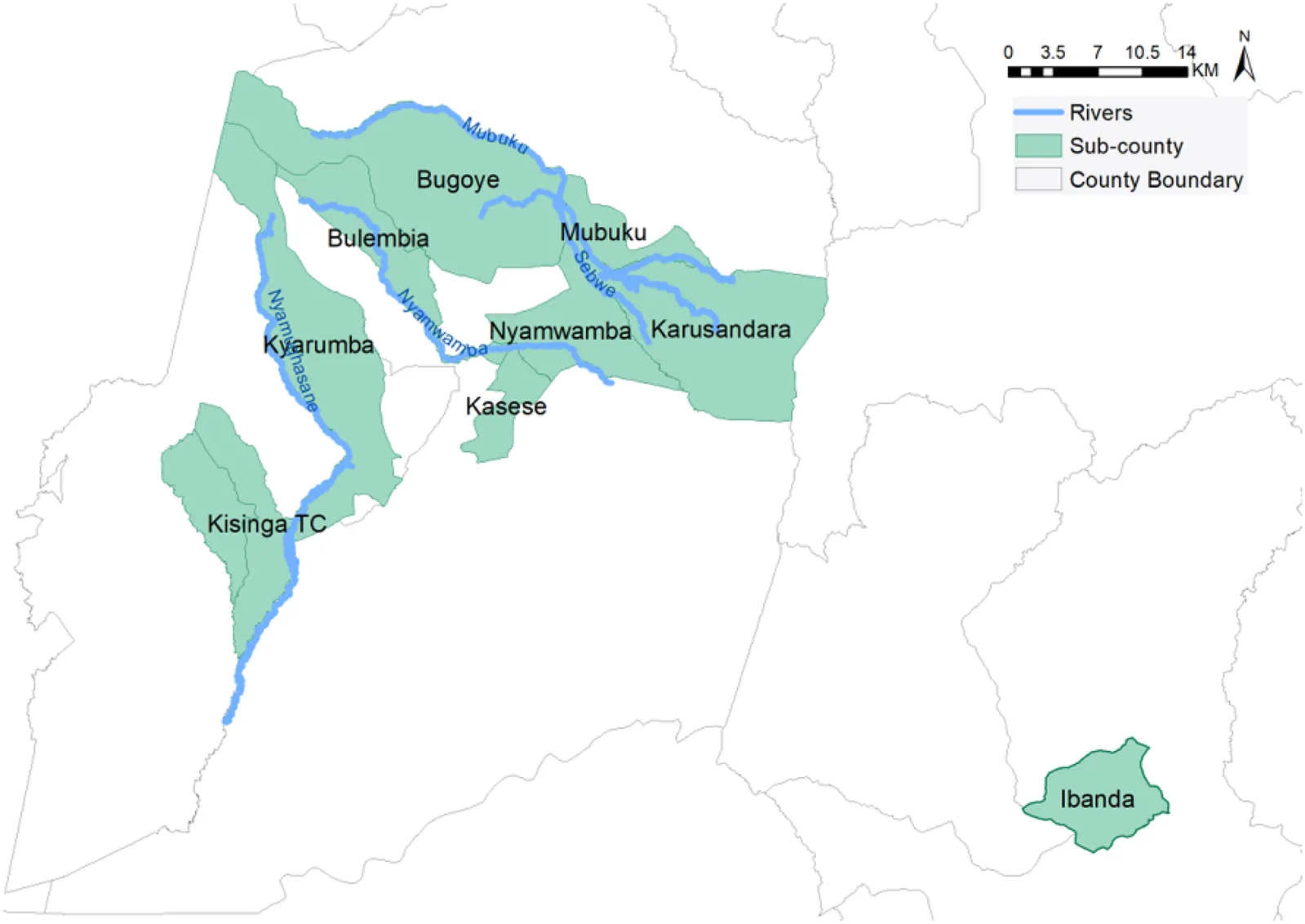
Map showing the location of the study settings, Kasese district

**Figure 2:**
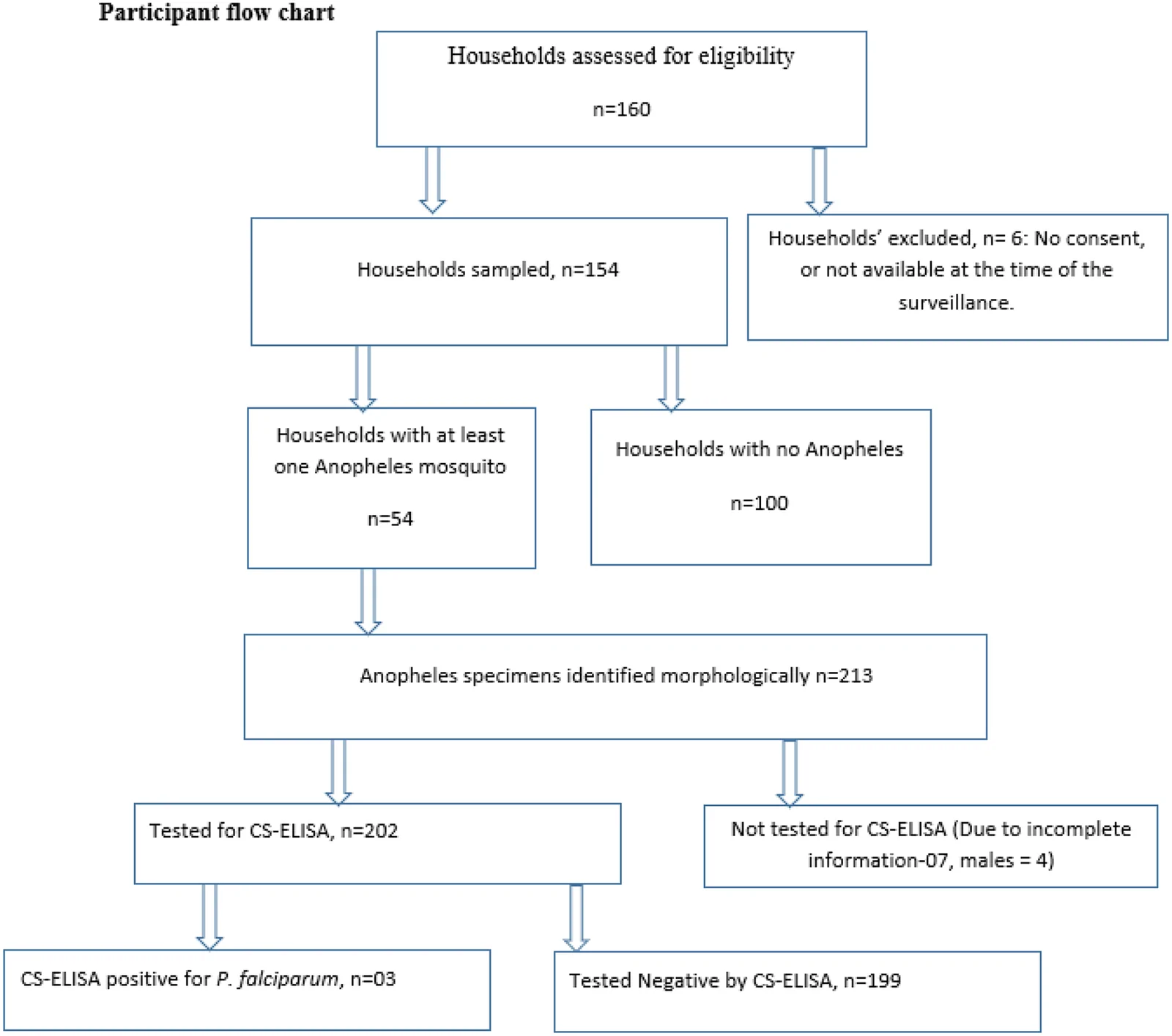
Participant flow chart

**Table 1:** Distribution of adult mosquito genera by household-Level covariates

Variable	Total Households	Adult Mosquitoes		Statistical test	p-value
	N = 154	Absent	Present		
		N = 18	N = 136		
		11.7%(95% CI: 7.5–17.9)	88.3% (95% CI: 82.2–92.5)		
**Place of residence (Sub-county)**				Fisher’s exact	0.09
Bugoye	12 (7.8%)	3 (25%)	9 (75%)		
Bulembya	7 (4.5%)	0 (0%)	7 (100%)		
Ibanda Town Council	24 (15.6%)	1 (4%)	23 (96%)		
Karusandara	26 (16.9%)	3 (12%)	23 (88%)		
Kasese municipality	9 (5.8%)	3 (33%)	6 (67%)		
Kasinga Town Council	4 (2.6%)	0 (0%)	4 (100%)		
Kisinga Town Council	35 (22.7%)	3 (9%)	32 (91%)		
Kyarumba	5 (3.2%)	1 (20%)	4 (80%)		
Kyarumba Town Council	15 (9.7%)	4 (27%)	11 (73%)		
Nyamwamba Division	17 (11.0%)	0 (0%)	17 (100%)		
**Catchment area (River)**				Fisher’s exact	0.95
Mubuku	59 (38.3%)	6 (10%)	53 (90%)		
Nyamugasani	59 (38.3%)	8 (14%)	51 (86%)		
Nyamwamba	36 (23.4%)	4 (11%)	32 (89%)		
**Sub-county distance from the riverbank**				Chi-square	0.06
Near (< 2km)	79 (51.3%)	13 (16%)	66 (84%)		
Far (> 2 km)	75 (48.7%)	5 (7%)	70 (93%)		
**Terrain**				Fisher’s exact	0.53
Low (< 1,000m)	67 (43.5%)	10 (15%)	57 (85%)		
Moderate (1,000m – 1,500m)	40 (26.0%)	4 (10%)	36 (90%)		
High (> 1,500m)	47 (30.5%)	4 (9%)	43 (91%)		
**Number of people in a household**				Fisher’s exact	0.88
1–3	20 (13.0%)	3 (15%)	17 (85%)		
4–6	87 (56.5%)	10 (11%)	77 (89%)		
>6	47 (30.5%)	5 (11%)	42 (89%)		
**Household ownership of LLNs**				Chi-square	0.58
No	78 (50.6%)	8 (10%)	70 (90%)		
Yes	76 (49.4%)	10 (13%)	66 (87%)		
**Number of LLINs in a household**				Fisher’s exact	0.42
1–2	41 (54%)	7 (17%)	34 (83%)		
3–4	28 (37%)	3 (11%)	25 (89%)		
≥5	7 (9%)	0 (0%)	7 (100%)		
**Age LLIN (Years)**				Fisher’s exact	0.36
Less than one year old	11 (14%)	0 (0%)	11 (100%)		
More than 2 years old	17 (22%)	3 (18%)	14 (82%)		
One to two years old	48 (63%)	7 (15%)	41 (85%)		
**History of flooding over the past year**				Fisher’s exact	0.13
No	91 (59.1%)	14 (15%)	77 (85%)		
Yes	63 (40.9%)	4 (6%)	59 (94%)		
**Main source of drinking water for the household**				Fisher’s exact	0.87
Piped water	93 (60.4%)	10 (11%)	83 (89%)		
Surface water (river, lake, stream, etc.)	42 (27.3%)	6 (14%)	36 (86%)		
Tube well or borehole	19 (12.3%)	2 (11%)	17 (89%)		
**Location of water source**				Fisher’s exact	0.19
Elsewhere	106 (68.8%)	15 (14.2%)	91 (85.8%)		
In one’s own dwelling	48 (31.2%)	3 (6.3%)	45 (93.8%)		
**Presence of window**				Chi-square	0.45
No	40 (26.0%)	6 (15.0%)	34 (85.0%)		
Yes	114 (74.0%)	12 (10.5%)	102 (89.5%)		
**Screened windows**				Fisher’s exact	0.06
No	67 (58.8%)	4 (6%)	63 (94%)		
Yes	47 (41.2%)	8 (17%)	39 (83%)		
**Presence of ventilators**				Chi-square	0.92
No	53 (34.4%)	6 (11.3%)	47 (88.7%)		
Yes	101 (65.6%)	12 (11.9%)	89 (88.1%)		
**Screened ventilators**				Fisher’s exact	0.25
No	81 (80.2%)	8 (10%)	73 (90%)		
Yes	20 (19.8%)	4 (20%)	16 (80%)		

**Table 2:** Distribution of adult *Anopheles* mosquitoes by household-level covariates

Variable	Total household	Adult Anopheles mosquitoes		Statistical test	p-value
	N = 154	Absent	Present		
		N = 100	N = 54		
		64.9% (95% CI: 57.0–72.1)	35.1% (95% CI: 27.9–43.0)		
**Place of residence(Sub-county)**				Fisher’s exact	0.45
Bugoye	12 (7.8%)	9 (75%)	3 (25%)		
Bulembya	7 (4.5%)	4 (57%)	3 (43%)		
Ibanda Town Council	24 (15.6%)	17 (71%)	7 (29%)		
Karusandara	26 (16.9%)	11 (42%)	15 (58%)		
Kasese municipality	9 (5.8%)	7 (78%)	2 (22%)		
Kasinga Town Council	4 (2.6%)	2 (50%)	2 (50%)		
Kisinga Town Council	35 (22.7%)	25 (71%)	10 (29%)		
Kyarumba	5 (3.2%)	3 (60%)	2 (40%)		
Kyarumba Town Council	15 (9.7%)	10 (67%)	5 (33%)		
Nyamwamba Division	17 (11.0%)	12 (71%)	5 (29%)		
**Catchment area (River)**				Chi-square	0.75
Mubuku	59 (38.3%)	36 (61%)	23 (39%)		
Nyamugasani	59 (38.3%)	40 (68%)	19 (32%)		
Nyamwamba	36 (23.4%)	24 (67%)	12 (33%)		
**Sub-county distance from the riverbank**				Chi-square	0.44
Near (< 2km)	79 (51.3%)	49 (62%)	30 (38%)		
Far (> 2 km)	75 (48.7%)	51 (68%)	24 (32%)		
**Terrain**					0.52
Low (< 1,000m)	67 (43.5%)	40 (60%)	27 (40%)		
Moderate (1,000m – 1,500m)	40 (26.0%)	28 (70%)	12 (30%)		
High (> 1,500m)	47 (30.5%)	32 (68%)	15 (32%)		
**Number of people in a household**				Chi-square	0.34
1–3	20 (13.0%)	15 (75%)	5 (25%)		
4–6	87 (56.5%)	58 (67%)	29 (33%)		
>6	47 (30.5%)	27 (57%)	20 (43%)		
**LLNs ownership**				Chi-square	0.91
No	78 (50.6%)	51 (65%)	27 (35%)		
Yes	76 (49.4%)	49 (64%)	27 (36%)		
**Number of LLINs in a household**				Fisher’s exact	**0.03**
1–2	41 (54%)	31 (76%)	10 (24%)		
3–4	28 (37%)	16 (57%)	12 (43%)		
≥5	7 (9%)	2 (29%)	5 (71%)		
**Age LLIN (Year)**				Fisher’s exact	**0.04**
Less than one year old	11 (14%)	10 (91%)	1 (9%)		
More than 2 years old	17 (22%)	13 (76%)	4 (24%)		
One to two years old	48 (63%)	26 (54%)	22 (46%)		
**Holes in LLIN**				Chi-square	0.61
No	23 (30%)	16 (70%)	7 (30%)		
Yes	53 (70%)	33 (62%)	20 (38%)		
**History of flooding over the past year**				Chi-square	0.12
No	91 (59.1%)	64 (70%)	27 (30%)		
Yes	63 (40.9%)	36 (57%)	27 (43%)		
**Main source of drinking water for the household**				Chi-square	0.44
Piped water	93 (60.4%)	64 (69%)	29 (31%)		
Surface water (river, lake, stream, etc.)	42 (27.3%)	25 (60%)	17 (40%)		
Tube well or borehole	19 (12.3%)	11 (58%)	8 (42%)		
**Location of water source**				Chi-square	1.00
Elsewhere	106 (68.8%)	69 (65.1%)	37 (34.9%)		
In one’s own dwelling	48 (31.2%)	31 (64.6%)	17 (35.4%)		
**Ownership of livestock, or other farm animals**				Chi-square	0.48
No	60 (39.0%)	41 (68%)	19 (32%)		
Yes	94 (61.0%)	59 (63%)	35 (37%)		
**Sharing houses with animals**				Chi-square	0.72
No	126 (81.8%)	81 (64.3%)	45 (35.7%)		
Yes	28 (18.2%)	19 (67.9%)	9 (32.1%)		
**Presence of window**				Chi-square	0.71
No	40 (26.0%)	25 (62.5%)	15 (37.5%)		
Yes	114 (74.0%)	75 (65.8%)	39 (34.2%)		
**Screened windows**				Chi-square	0.97
No	67 (58.8%)	44 (66%)	23 (34%)		
Yes	47 (41.2%)	31 (66%)	16 (34%)		
**Presence of the eaves in the living room**				Chi-square	0.74
No	138 (89.6%)	89 (64.5%)	49 (35.5%)		
Yes	16 (10.4%)	11 (68.8%)	5 (31.3%)		
**Screened eaves**				Fisher’s exact	0.55
No	12 (75%)	9 (75%)	3 (25%)		
Yes	4 (25%)	2 (50%)	2 (50%)		
**Presence of ventilators**				Chi- square	0.88
No	53 (34.4%)	34 (64.2%)	19 (35.8%)		
Yes	101 (65.6%)	66 (65.3%)	35 (34.7%)		
**Screened ventilators**				Chi-square	0.31
No	81 (80.2%)	51 (63%)	30 (37%)		
Yes	20 (19.8%)	15 (75%)	5 (25%)		

**Table 3:** Adult *Anopheles* mosquito species identified across the study area

*Anopheles* species	Number of mosquitoes (Frequency, n = 213)	*Anopheles* species (Percentage)
*An. gambiae s.l*	157	73.7
*An. funestus*	20	9.4
*An. pretoriensis*	17	8.0
*An. rivulorum*	07	3.3
*An. mouchetti*	02	0.9
*An. nili*	01	0.5
*An. marshalli*	2	0.9
Un identified	7	3.3
Total	**213**	**100**

**Table 4:** Abundance of adult *Anopheles* mosquito species by sub-county

Sub county	*Anopheles* mosquito	Number of mosquitoes	*Anopheles* species collected (%)	Total *Anopheles* collected (%)
Bugoye	*An. pretoriensis*	2	40	2.5
	*An. funestus*	2	40	
	*An. rivulorum*	1	10	
Sub total		**5**		
Ibanda Town Council	*An. gambiae s.l*	7	53.9	6.4
	*An .funestus*	2	15.4	
	*An. pretoriensis*	1	7.7	
	*An. rivulorum*	2	15.4	
	*Unidentified*	1	7.7	
Sub total		**13**		
Karusandara	*An. gambiae s.l*	84	87.5	47.3
	*An. pretoriensis*	4	4.2	
	*An. mouchetti*	2	2.1	
	*An. nili*	1	1.0	
	*An. rivulorum*	1	1.0	
	Un identified	4	4.2	
Sub total		**96**		
Bulembya	*An. gambiae s.l*	06	66.7	4.4
	*An. funestus*	01	11.1	
	*An. pretoriensis*	02	22.2	
Sub total		**9**		
Kasinga TC	*An. gambiae s.l*	13	86.7	7.4
	*An. funestus*	2	13.3	
Sub total		**15**		
Kisinga TC	*An. gambiae s.l*	13	65	9.9
	*An. funestus*	6	30	
	*An. pretoriensis*	1	5.0		
Sub total		**20**			
Kyarumba	*An .gambiae s.l*	3	23.1	6.4
	*An. funestus*	4	30.8	
	*An. pretoriensis*	5	38.5	
	*An. rivulorum*	1	7.7	
Sub total		**13**		
Kyarumba Town Council	*An. funestus*	03	37.5	3.8
	*An. gambiae s.l*	01	12.5	
	*An. pretoriensis*	01	12.5	
	*An. marshalli*	02	25	
	*Un identified*	01	12.5	
Sub total		**8**		
Kasese Municipality	*An. gambiae s.l*	4	100.0	2.0
Sub total		4		
Nyamwamba Division	*An. gambiae s.l*	26	86.7	14.8
	*An. pretoriensis*	1	3.3	
	*An. rivulorum*	2	6.7	
	Un identified	1	3.3	
Sub total		**30**		
Grand Total		**213**		

**Table 5:** Abundance of adult *Anopheles* mosquito species across river catchments

River catchment	*Anopheles* species identified	Number of mosquitoes collected	*Anopheles* species collected (%)	Proportion of Anopheles collected (%)
Mubuku	*An. pretoriensis*	7	6.4	51.6
	*An. rivulorum*	3	2.7	
	*An. gambiae s.l*	88	80	
	*An. funestus*	4	3.6	
	*An. mouchetti*	2	1.8	
	*Un identified*	6	5.5	
	**Total**	**110**		
Nyamugasani	*An. gambiae s.l*	30	53.6	26.3
	*An. funestus*	15	26.8	
	*An .pretoriensis*	7	12.5	
	*An. marshalli*	2	3.6	
	*An. rivulorum*	1	1.8	
	*Un identified*	1	1.8	
	**Total**	**56**		
Nyamwamba	*An. gambiae s.l*	39	83.0	22.1
	*An. pretoriensis*	3	6.4	
	*An. funestus*	1	2.1	
	*An. rivulorum*	3	6.4	
	*An. nili*	1	2.1	
	**Total**	**47**		
Grand total		**213**		

## Data Availability

The individual data that supports the results will be shared following publication provided the investigator who proposes to use the data has approval from an Institutional Review Board (IRB), Independent Ethics Committee (IEC), or Research Ethics Board (REB), as applicable, and executes a data use/sharing agreement with UNC.
